# Dermatology residents as educators: a qualitative study of identity formation

**DOI:** 10.1186/s12909-023-04186-4

**Published:** 2023-03-30

**Authors:** Lulu Alwazzan, Ruaa AlHarithy, Hend M Alotaibi, Thuraya Kattan, Monira Alnasser, Taif AlNojaidi

**Affiliations:** 1 Imam Mohammad Ibn Saud Islamic University, Riyadh, Saudi Arabia; 2 Princess Nourah Bint Abdulrahman University, Riyadh, Saudi Arabia; 3 King Saud University, Riyadh, Saudi Arabia; 4 Saudi Commission For Health Specialties, Riyadh, Saudi Arabia; 5 King Fahad Medical City, Riyadh, Saudi Arabia

**Keywords:** Dermatology, Residency, Medical education, Health professions education, Continuous professional development, Professional development, Resident as teacher, Resident as educator, Residency competencies, Curriculum

## Abstract

**Background:**

One of the many identities a physician comes to form during their career is their identity as an educator. Exploring formation of this identity may enrich our understanding of how physicians make decisions related to their roles as educators, their behaviors, and how this ultimately influences the educational environment. It is the aim of this study to investigate educator identity formation of dermatology residents while early in their careers.

**Methods:**

Drawing on a social constructionist paradigm, we conducted a qualitative study, utilizing an interpretative approach. We examined longitudinal data over a 12-month period using dermatology residents’ written reflections from their professional portfolios and semi-structured interviews. We collected this data as we progressed through and beyond a 4-month professional development program designed to encourage residents’ growth as educators. Sixty residents in their second, third, or final year of residency programs located in Riyadh, Saudi Arabia were invited to take part in this study. Twenty residents participated with sixty written reflections and 20 semi-structured interviews. Qualitative data were analyzed using a thematic analysis approach.

**Results:**

Sixty written reflections and 20 semi-structured interviews were analyzed. Data was categorized according to themes corresponding to the original research questions. For the first research question regarding identity formation, themes included definitions of education, the process of education, and identity development. For the second research question, 1 theme entitled professional development program included, the following sub-themes: individual act, interpersonal activity, and an organizational undertaking, with many believing that residency programs should prepare residents for their educator roles. Participants also described newfound leadership ambitions of creating new dermatology fellowship programs as a result of taking part in the Resident-as-Educator program.

**Conclusions:**

Our study provides insights on the dynamic formation of educator identities amongst dermatology residents. Investment in developing residents as educators through professional development programs may instigate transformational change on the individual physician level and profession’s level.

**Supplementary Information:**

The online version contains supplementary material available at 10.1186/s12909-023-04186-4.

## Introduction

The professional development of a physician-in-training has recently expanded to include their role as an educator [1, 2]. Scholars in this area have long advocated for engaging physicians at an early stage and during postgraduate training in their roles as educators, believing that residents enacting this role benefited the medical students, other residents, the residents themselves (increasing self-awareness and greater job satisfaction) and the system [3–6]. However, despite the recognition, development of residents in their capacity as educators remains elusive.

One reason for the lag could be residency education’s focus on the clinical aspect of a physicians’ development, making less room for other capacities such as education, research and leadership. Often such capacities are considered inherent in clinical practice and not in need of structure and active development. Over time this belief has been leveled by the call for more nuanced understandings of a physician’s training and the development of educational frameworks such as the CanMeds [7]. In addition to the medical expert, the CanMeds framework includes 6 competencies every physician should master including: physician as professional, communicator, collaborator, leader, health advocate, and scholar. What concerns us is the latter, the role of a scholar which is described in the CanMeds as a “commitment to lifelong learning, teaching others, and contributing to scholarship” [8].

Second, while many recognize the importance of building such capacities, program design remains challenging. The literature reports several professional development programs that target residents’ educational knowledge and skills [1, 3, 9–12]. However, these programs focus on arming residents with skills to perform current teaching duties (e.g. small group teaching), neglecting future more pertinent performances as educators (e.g. expanding educational programs).

To understand more deeply the impact of such development programs, scholars have called on the study of identity formation [13]. How one forms a professional identity in and through these programs may aid in understanding how one behaves in certain situations in the workplace [14] and how that may influence the educational environment. Exploring the way in which dermatology residents, physicians at the beginning of their careers, come to form identities as educators is crucial to our understanding of their development. Such identity formation may unveil how residents think of themselves as educators and how they go about enacting their roles as educators, what decisions they make that influence their professional growth, as well as how they influence the educational environment for others such as medical students, peers, and the system. It is for these reasons that this study begins by asking: 1) How do dermatology residents form identities as educators? 2)What is the role of professional development programs in their growth as educators?

## Background

### Identity formation

Although several orientations exist in the literature about how identities may be examined and researched, [15] in this study a social constructionist stance is taken. The definition of identity formation as the development of professional values, actions, and aspirations [16] in a co-constructed manner through interactions with others and the environment dynamically and fluidly was adopted [15]. To understand identity one must examine not only *what* identities there are but *how* they come to be, this can be done through the examination of narrative and language. Furthermore, it is important here to differentiate identity from role [2]. Identity is how one defines themselves (e.g. I’m an educator), while role is what is expected of one to perform (e.g. I teach at a university). Negligence of identity development alongside expectations of role enactment (e.g. resident must teach students and peers) may lead to incongruence between one’s multiple professional identities (e.g. to be a clinician and tend to patients in the clinic is more important than being an educator and teaching the medical students during). It may also have several negative outcomes such as identity dissonance, leading to uncertainty and emotional difficulties, role neglect, eventually this may lead to burnout.

Such tensions were explored in medical education in several undergraduate contexts [1, 2, 15, 17–21] and amongst *practicing* health professionals, exploring the formation of professional identities beyond the identity of the clinician, their identities as educators and leaders [11, 22–24]. However, while a few studies exist on the clinician identity formation for residents [25], to our knowledge, no studies explore educator identity formation amongst this group.

### Professional development

Past studies in professional development have focused primarily on the design and implementation of short-term programs. Such programs are often targeting more mature healthcare professionals and not those physicians in training [26]. Although residents and medical students often take part in such programs, it is not clear how beneficial they are to physicians-in-training.

Less progress has been made on defining and classifying what is considered ongoing professional development activities for residents. Some studies draw attention to the needed structured support to ensure transformative professional change amongst residents [27, 28]. To prepare residents for their educator role in particular, various programs were conducted [3, 4, 29]. The impact of such educational interventions remains to be seen.

## Conceptual framework

To deepen understanding further, Kelchterman’s [30] conceptual model of professional identity was adopted. The model highlights the influence of the professional self on professional behaviors according to two dimensions: *retrospective* and *prospective.* The former includes the concept about self as one examines the past and the latter includes the concept about self as one examines the future. The retrospective dimension comprises four components: (1) Self-image: The general principles governing one’s professional behavior; (2) Self-esteem: Valuing and evaluating oneself as a professional, involving judgment of performance; (3) Job motivation: Motives behind entering and staying in the profession and commitment towards it; (4) Task perception: How an individual defines the work, professional relationships, and behaviors. As for the prospective dimension, it is made up of one component, future perspectives. It highlights how professionals see their future career progression, opportunities, and development.

## Method

### Study Design

This study is based on a social constructionism epistemology, where knowledge is thought to be socially situated and based on human practices as co-constructed in-between individuals. Aligned with an interpretative approach, written reflections (written by the resident) were collected, and semi-structured interviews (using narrative interviewing techniques) were conducted. From the collected data residents’ views and experiences of education within the social context of the dermatology residency program were examined deeply. (See Table 1). The Institutional Research Board at the Security Forces Hospital in Riyadh, Saudi Arabia granted the study ethical approval.

**Table 1:** Study design

Epistemology	Research design	Research method	Data analysis
Social constructionism	Interpretive inquiry	Written reflections (resident narratives) Semi-structured interviews	Thematic analysis

### Subject, setting, and sampling

The target population were 60 residents enrolled in the dermatology residency program in the Riyadh Region at the time the study was conducted between 2019 and 2021. Year 1 residents were excluded from the study as they spend their first residency year rotating in other specialties: pediatrics and internal medicine and were not obligated to attend professional development programs. Given the small population (n = 60), we conducted an email campaign to recruit all residents, asking for permission to include the written reflections and for a one-on-one interview. Twenty agreed to participate. Not all residents committed to the monthly written reflections. Ultimately, we collected 60 reflections from the 20 willing participants and these participants were interviewed as well. To the knowledge of the authors, residents did not receive formal training in medical education prior to the proposed program.

### Resident-as-Educator Program

The curriculum of the Resident-As-Educator Program was designed by the lead author and facilitated by the co-authors during the monthly half-academic days. The program included 4 workshops on the following topics: Becoming an educator, Fulfilling the teacher role, How is knowledge made, and Education: a team sport. Workshops were held once a month. Each workshop was 2 hours long. The list of topics was created based on a literature review of other residents as teacher curricula [4, 31, 32] and were found to be relevant to residents’ experience as educators (See Appendix A for program curriculum).

The workshops included 10-minute didactic lectures followed by small group discussions and then a large group discussion that included everyone. These teaching strategies aided us in engaging residents in deliberate practice and reflection during the workshops. Facilitators were experts in the content area specific to the session. Kirkpatrick’s Evaluation Model [33] was used to orient the assessment of learners (See appendix A for further details). Resident were formatively assessed through oral reflections during workshops and summatively through written reflections 1 month after each workshop. Moreover, residents took part in oral presentation at the end of the program. Residents were also afforded the opportunity to reflect during the one-on-one interviews conducted 6–12 months after the program was completed.

### Data collection

There were 2 sources of data for this study, 60 written reflections and 20 semi-structured interviews. The written reflections were submitted as part of residents’ professional portfolios. Those included 1 reflections collected within a month after the module was conducted and prompted residents with questions discussed during the module discussions (See Table 2 for prompts). In total, each resident wrote 2–4 reflections.

**Table 2:** Reflection prompts

Module	Written reflection prompt
Becoming an educator	Who am I as an educator?
Fulfilling the teacher role	How are we being taught in dermatology residency education?
How is knowledge made?	Where does knowledge in the field of dermatology come from?
Education: A team sport	How do you teach to and with others?

The semi-structured in-depth interviews were conducted in English using a narrative interview technique and using an interview guide developed for this study, according to research objectives and in alignment with Kelchterman’s Model (See appendix B: for interview guide). Questions included what does education mean to you? How do you educate others? And how is residency education different from previous stages of your education? Participants were allowed to express themselves freely and prompts were used to engage participants further and only when needed. Participants were invited via email. The semi-structured interviews lasted 20–60 min and were conducted via ZOOM or telephone. The study purpose and interview procedure were explained to the participants and verbal and written permission were sought (See appendix B for interview procedure). Participants were asked to sign a consent form. LA and TA conducted the interviews and audio-recorded them. Each interview was transcribed by a professional. Collected data was stored electronically, in a hard drive accessible only by the primary investigator and her co-investigators.

### Data analysis

Drawing on a social constructionist approach [34] and using a Thematic analysis framework, the collected data was analyzed. A social constructionist approach emphasizes that individuals construct the world around them and understand it through the experiences they have with others and the environment [35–37]. Such an underpinning allows for an interpretative exploration, thus there is no a priori theory, rather the data guided the exploration.

Thematic analysis is a qualitative research approach that allows for residents’ views and experiences to be interpreted within the context of their residency program. This approach can aid our understanding of an individual’s experience and how they represent themselves to others [35]. Themes were developed according to an initial data set of 2 reflections and 2 interview transcripts by two different researchers independently. Each researcher, went through the two reflections and 2 interview transcripts, reading and familiarizing themselves with the data. At this stage, each researchers coded sentences and whole paragraphs by writing initial descriptions e.g. education experiences. Both researchers then met to discuss their initial descriptions and illustrative quotes. Through discussion, the two authors refined the names of the codes and further categorized each into sub-themes. After which, an analysis framework (See Appendix C) was developed and used to analyze the rest of the data. The analysis framework was continuously refined by ongoing discussions amongst the rest of the team members. Data analysis was done through Atlas.Ti, a qualitative analysis software (Berlin, Germany).

## Results

The data revealed rich insights into educator identity formation amongst dermatology residents. Overall, 20 participants took part in the study in years 2, 3, 4 of the 4-year residency program. Seventeen participants were female and 3 were male. Based on the 60 written reflections and 20 interviews, themes were broadly categorized according to research question. The first research question comprised of participants’ views and experiences of education and educational practice in medicine. These themes include definitions of education, the process of education, and the various identities formed by residents. For the second research question regarding professional development programs one theme is highlighted. Appendix C: Analysis Framework includes the cascade of research questions, themes, sub-themes, their definitions, and number of found quotes in each sub-theme. In what follows, the themes are organized according to our research questions.

### How do dermatology residents form identities as educators?

#### Defining education

In response to the question ‘Who am I as an educator?’ participants wrote in their personal professional portfolios and shared during the interviews: definitions of education, attributes of a good educator, and their aspirations of becoming a better educator. Some residents defined education within the confines of the residency program:

“So as part of our residency program we’re kind of obligated to give lectures that are carried out as part of the training. Those lectures are either joint or within the hospital that we rotate in. This is one of our roles as educators.” (Resident 5_Interview)

Another participant defined education more holistically, drawing attention to the environment:

“I mean, to me, it’s the cultivating, supportive, and professional relationship you have. The encouragement of self-inquiry and research and reflective activity is going to culminate into allowing you to be part of this educator establishment or educational establishment. In my career, I don’t think I– it’s not the process of teaching itself. It’s the entire environment you’re in” (Resident 8_Interview)

Another participant defined education beyond residency:

“I’m the eldest of the family among my brothers and sisters. So I always try to share my experiences with my brothers and sisters…give advice. I’m always willing to help out with their studies if someone has difficulty understanding a certain topic. My younger sister was enrolled in medical school for a while, so I was helping her out with that” (Resident 9_Interview)

Some participants described attributes of being a successful educator, suggesting that being a good educator, required committing to lifelong learning, having an ability to design and communicate:

“In my opinion, to be a successful educator, a person needs to be a lifelong learner in the first place, a *designer* who’s able to simplify and deliver information using creative teaching methods and lastly a good *communicator* who can discuss and view knowledge from different perspectives” (Resident 13_Written reflection)

Participants reflected on their aspirations of becoming better educators by engaging in informal activities with juniors. These authentic personal experiences, as reported by participants, helped them become better learners:

“Yes. So I do group studies, and most of the time, I’ve been teaching more junior residents, and I found it really helpful for me, more than the residents themselves…I’ll never forget it if I give it to someone or if I explain it.” (Resident 15_Interview)

#### The process of education

Participants reflected on how they went about educating others. One participant conceptualized education as prioritizing and guiding learners to appropriate learning resources:

“Sharing important resources and highlighting important topics is more important than offering a piece of information that might be forgotten at the end of the day.” (Resident 17_Written reflection)

Some participants spoke of education as an act of paying it forward, as a resident shared:

“because if you get benefits from those sessions from the people who are senior to you at that time, it’s like a mandate in a way to give it to the juniors.” (Resident 12_Interview).

The process of education was also viewed as context-based: in the clinical setting or in the lecture hall.

“Being an R3 resident we’re also sharing information *during clinics* with our colleague residents…This is my role as an educator.” (Resident 20_Interview)

#### Educator identity formation

In written reflections and in some of the early interviews, participants struggled with their identity as an educator as one participant shared:

“Okay. Actually, I don’t find myself that much as an educator. But I have things I did in my med school, also during residency. We did summaries, we did lectures, we did seminars, we did also some lectures during our residency. Those are the things that I did, and I don’t find them that much for a purpose as education. I think I have to fulfill all the things that I need, then I can be educator. Uh-huh. So– Yeah, I have to gain all the knowledge I need, then, I can deliver it to people” (Resident 15_Interview)

While overtime and during interviews conducted months after the program, other residents began to form educator identities in several multifaceted ways:

##### Educator as leader

“After the program, I have ambitions of creating more dermatology programs. I can do it, now that I know about the need. We don’t have many (programs) in Saudi Arabia” (Resident 13_Interview)

##### Educator as simplifier

“Some people tell me that I’m good at teaching. I try to simplify ideas as much as I can. I’ve had the sort of feedback before that I simplify what people find difficult. And people usually tend to come to me to explain certain things that they find difficult. However, I don’t think I’m an excellent educator. I’m still working on that aspect, but I’m always willing to help.” (Resident 13_Interview).

##### Educator as knowledge curator

In the following quote, the participant forms their identity as an educator, and speaks using plural pronouns:

“*We* do a formal topic review, which is *our* Tuesday activity. So you’ve been assigned to a certain topic, and you’re going to read through this topic from different resources, and then you’re going to approach it in a way where it’s presentable and simplified for your colleagues to learn about and to know. This is the formal aspect of it. But then you have the broader part of this process which is the journal club reviews, for example, or the grand rounds. This is the time to challenge and to explore. So if it’s the journal club, you’re going to decide which, for– you’re going to be given options to read, for example, from different journals out there, and then you’re going to choose a topic of interest, and you’re going to explore that. So you’re going to learn how to– this is your own learning experience. Then you’re going to relay it to the rest.” (Resident 19_Interview).

##### Educator as community advocate

In the following quote, one resident shares the positive influence of being a part of a community:

“As an educator, I love doing teaching sessions because it gives me a positive feeling. It gives me motivation. It gives me a feeling that I helped my community; I helped my colleague. And it’s like retaining the feeling” (Resident 6_Written reflection).

##### Educator as self-motivated

One resident explained how he self-motivated:

“Of course self-motivation is one of the cards as a teacher that helped me encourage myself before giving any session to any group, I motivate myself saying that: you can do it! This is a small group.” (Resident 16_Interview).

##### Educator as learner

In the following quote, a participant shared the need to recognize being a learner in order to be a good educator:

“You need to have an open-minded approach to learning. As a learner to be able to educate from anything that is surrounding you. So it is a process. It’s not something that you can take. It’s not a certificate that you can just work for. It’s a lifelong process of learning.” (Resident 17_interview).

##### Educator as collaborator

In the following quote the participants highlight the role of reciprocity in education:

“It’s always a two-way street. You need the other person, the recipients to want to learn or to want to be good teachers. If you teach a person who doesn’t want to be taught, you’re going to waste your time. It’s going to have to be a two-way street.” (resident 9_Interview).

## What is the role of professional development programs in residents’ development?

Participants agreed that developing their educator knowledge, skills, and attitudes is a continuous endeavor that must be tended to. Participants viewed their professional development as an individual lifelong effort, an interpersonal effort, and as a structured part of residency education. As an individual effort, one participant shared:

“As an educator I am trying to develop my skills continuously, I try to teach other residents, interns during the clinic simple basic things, as well as I try to encourage them to read interesting things related to the cases we see.” (Resident 14_Interview)

Another sub-theme found was *collegiality in becoming an educator*, as one senior resident came to share:

“When you trust your colleagues it makes the process of learning and working together more enjoyable.” (Resident 14_Reflection)

Finally, the role of the residency program and the larger field of dermatology in the development of residents as educators, one participant shared:

“Several fields (Medical specialties) excel in growing educators by permitting them chances and opportunities, but for us we don’t have the proper skills sometimes” (Resident 13_Interview)

Another participant looked to the future and that residency programs should take a more active role in in the development of residents as educators:

“Well, as a resident we don’t get a lot of opportunities to be educators. But, thankfully, in our program, we get to present to other residents. Continuous practice of teaching and learning and professional development will help healthcare providers build excellent educators. Hopefully, residency training will pave the way for me to develop new skills and grow as an educator” (Resident 12_Interview).

## Discussion

Our longitudinal analysis shows the complex evolving way in which these themes interact as residents formed their educator identities over the 16-month period. Oriented by our chosen conceptual framework: Kelchterman’s [30] conceptual model of professional identity, we found that self-motivation, positive teaching and learning experiences during residency, and an encouraging culture in the profession of dermatology influenced that development of self-image and the formation of a professional educational identity. Participants described both intrinsic and extrinsic motivators in their roles as educators. Based on their residency experience, participants reported a positive impact on personal and professional development. This development included increased confidence, improvements in practice and identification as educators by themselves and others.

Participants reflected on their educator identity retrospectively by contemplating their past experiences and producing definitions of education and the process of education. Some participants defined education in a broader sense, considering experiences during medical school while peer teaching and in their personal lives. Participants also defined education within the confines of residency education, indicating that the residency program itself was an encouraging factor for engaging further as an educator. This is aligned with Kelchterman’s [30] *task perception*, defining their work and educational professional relationships, whether with juniors or seniors to be collegial in nature, identifying teaching and learning as a shared responsibility.

### Professional development

Many of our participants framed their engagement in educational activities as teachers as a form of professional development. Self-directed and peer learning were identified as two main ways residents went about preparing for their roles as educators. Both self-directed and peer learning are viewed as promoters of self-regulation that enhances deeper understanding and enhanced enactment of the teacher role [29]. For meaningful participation to take place, it seems participants depended on self-motivation and an internal desire to give to others and to give back to the field of dermatology. In the same instance, participants thought it necessary for formal training in medical education, citing the lack of know-how in contemporary principles of medical education. The literature suggests that the investment in formal training in medical education influences self-perception, participation in educational activity, and engagement in educational leadership roles [13, 38, 39] Although, no association was found between formal training and future engagement in education, [40] our findings allude to the need for a multipronged approach at the individual, residency program and profession, and the regulator levels. Moreover, our findings show a need for cultivating identity formation rather than mere role allocation for residents to engage more meaningfully in their roles as educators.

### Valuing oneself and evaluating oneself as an educator

Previous studies reported transformational change and a greater sense of empowerment and self-efficacy in educational practices as a result of competence in educational knowledge and skills [13, 38, 41]. In our study, residents reported empowerment as educators as a result of the feedback they received from juniors and seniors. This led to self-assessment and a desire to improve educational practices and to face challenges that may arise. According to our participants’ experiences, improvement as an educator leads to improvement as a clinician. Participants lacked the knowledge, skills, and attitudes adopted by current medical educators, being student-centered and having the knowledge of educational theory and providing feedback [42]. Moreover, they seemed less confident in assessing their performance as educators. This further emphasizes the need for professional development as educators for medical residents.

## Strengths and limitations

Coupling written reflections with interviews collected over a period allowed for a more nuanced study of identity formation amongst our participants, although data was self-reported. Self-reported data may be influenced by the resident’s position as a learner. Residents may have answered in what they thought to be desirable answers. The study allowed for the examination of contextual data; however, it was conducted in one geographical region and findings may not be applicable in varying regions in the Kingdom of Saudi Arabia. Furthermore, the study was limited because of the low number of interviews (20) from a program of 60 residents. A wider set may have yielded richer data, as residents experiences and development might be different, thus the generalizability of the study findings is limited. Another limitation was the low number of male participants. More female residents participated in this research. As a result, our findings might not reflect male residents’ views of their role as educators. Future studies must elicit the views and experiences of male medical residents. As this study was conducted prior to SARS-CoV-2 pandemic, it is not known how the reported program and indeed findings of the study would be different. It is anticipated that such professional development programs should include an e-learning component, for example how to teach using e-learning modalities. As a result of the pandemic, e-learning is now better embraced and utilized to make teaching and learning more efficient. Future iterations of this program may benefit from addressing this form of teaching and learning.

## Implications

Findings of this study suggest that dermatology residents are willing to enact their roles as educators. They also suggest that by paying attention to identity formation, the dermatology community may be better situated to prepare residents for their educator roles. By deliberate action and through structured training in educational principles, curriculum design, and assessment, a timely opportunity to develop residents in this aspect is possible. Formal longitudinal training opportunities allowed the construction and co-construction of meaning to take place between the self and the workplace community, this co-construction leads to identity formation [43]. Identities seem to develop in the active interplay between self motivation and the learning culture of the residency program. As a result, residents are better able to enact their educator roles more fully beyond postgraduate training, with prospects that include leadership positions such as program directorship and heads of departments. It is therefore recommended that formal training in medical education to be embedded within residency programs. Additionally, development of policies and procedures that support such programs is necessary.

The need for a positive culture around education and formal training opportunities may lead to a transformational change both at an individual level and at the level of the profession. Cultivating educator identities through professional development programs amongst dermatology trainees may facilitate such transformational change. Our findings offer a nuanced understanding of how educational identities can form and how training programs can cultivate this formation. Future research should pay close attention to educational context and how that influences educator identity development.

## Electronic supplementary material

Below is the link to the electronic supplementary material.

Supplementary Material 1

Supplementary Material 2

Supplementary Material 3

## Data Availability

The datasets used and/or analysed during the current study are available from the corresponding author on reasonable request.
