# Supplementary material for: Dermatology residents as educators: a qualitative study of identity formation
Source: BMC Med Educ. 2023 Mar 30;23:199. doi: 10.1186/s12909-023-04186-4 (PMC10061385; doi:10.1186/s12909-023-04186-4)
Supplement: Supplementary file 1 — Supplementary Material 1 [file 12909_2023_4186_MOESM1_ESM.docx]

**Resident as Educator**

Dermatology residency program

Riyadh Region

Dr Lulu Alwazzan

Dr. Roa’a Alharthey

Dr Hind Alotaibi

**1. Introduction:**

The following document is a pilot design of a professional development program within dermatology residency education. It is also an educational tool that raises awareness on best educational theory and practices. The first section, is a broad look at how the professional development (PD) program within the Dermatology residency program for the academic year 2019/2020 is to be conceptualized, followed by an explanation of some of the used educational terminology. The second section is a curriculum plan for the 4 different activities that will be held monthly. The activities are interdependent and build on one another. In the fifth month learners will be summatively assessed using written reflections and an oral presentation at the end of the program.

**1.1**

| **Program:** Dermatology resident as educator | | |
| --- | --- | --- |
| **Theoretical Paradigm** | **Purpose** | **Aims (Program -level intended learning outcome)** |
| Social Constructionism | To foster academic growth in the field of dermatology and to establish benchmarks for the running of professional development within residency education in the Saudi Arabian context. | To cultivate an educator’s identity amongst dermatology trainees. |
|  |  | To prepare dermatology trainees for their teaching roles. |
|  |  | To engage dermatology trainees in the knowledge production of the field. |
|  |  | To increase collegiality in the field of dermatology. |

**1.2 What is a theoretical paradigm?**

It is the set of beliefs that guides the way we do things. For the proposed program, we draw on social constructionism. A widely used theory of knowledge, social constructionism examines the development of co-constructed understandings of the world that come to form the basis for shared assumptions about the world. For the proposed CPD program within residency education, learners are encouraged to articulate their experiences and beliefs, and to come to a shared meaning with their fellow residents and facilitators. In the following curriculum plan, the reader will notice the developed aims, intended learning outcomes, and aligned proposed instructional strategy provide an orientation and learning environment that encourages the learners to make meaning of their experiences, all the while, becoming reacquainted with familiar concepts (e.g. Education).

**1.3 What is meant by *purpose*?**

Purpose is the answer to the question why are we doing what we are doing? In other words, it is the reason that motivates us to do something. For the proposed program, the purpose is to foster academic growth in the field. More deeply, the intent is to broaden the professional repertoire of dermatology residents. Such that, graduates of the dermatology program not only practice dermatology, but are engaged in aspects of knowledge production, dissemination, and translation.

**1.4 What are aims (program-level intended learning outcomes)?**

They are short statements that describe, broadly, what is to be achieved. Such statements put the onus on the program developer and implementer. Aims can be contrasted to the module-intended learning outcomes which put the onus on the learner; the short statements that spell-out what the learner should be able to do after the module has taken place, putting the onus on the learner.

**2. Modules**

| **2.1 Module 1:** Becoming an educator *(Kelcheterman’s model: Self-image and task perception)* | | | **130 minutes** | **November 2019** |
| --- | --- | --- | --- | --- |
| **Aim** | **Intended learning outcome (ILO)** | **Instructional design** | **Material** | **Assessment** |
| To cultivate an educator’s identity amongst dermatology trainees. (Self-image) | Introductions and signposting (20 minutes) | | | |
|  | Discuss your role as an educator. | **Small group activity (5 persons):** Each group brainstorms for 10 minutes then **large group discussion** 10 minutes. **Main point:** What is education? ***Prompts***: Educator vs teacher?  In the classroom vs on the internet?  Share an example of a good educator? Share an example of a bad educator?  How do you say education in Arabic?  **Brief didactic activity:** 10 minutes | Pop-up board and markers for each group  Powerpoint presentation/Projector | **Formative:** Give small group verbal feedback during discussions; give verbal group feedback on performance after large discussion.  Topic: Who am I as an educator?  **Summative (Last session):** Educator project. |
|  | Differentiate being an educator in dermatology community from educator in medical community (Horizontal growth). | **Small group activity (5 persons):** Each group brainstorms for 10 minutes then **large group discussion** 10 minutes. **Main point:** Do I change as an educator based on context? How?Why? ***Prompts***: Is an educator in dermatology different from the medical community? What are descriptors for educator in dermatology field versus medical field? Share an example of an educator in dermatology and broader medical community. |  |  |
|  | 10 minute break | | | |
|  | Contrast the chronological stages of being an educator (vertical growth). | **Small group activity (5 persons):** Each group brainstorms for 10 minutes then **large group discussion** 10 minutes. **Main point:** What are the stages of being an educator? ***Prompts***: as a medical student? A resident? A fellow? A consultant? At tertiary hospital? At university? In Saudi Arabia? Outside Saudi Arabia?  **Brief didactic activity:** 10 minutes | Pop-up board and markers for each group  Powerpoint presentation/Projector | **Formative:** Give small group verbal feedback during discussions; give verbal group feedback on performance after large discussion.  Topic: How are we being taught in dermatology residency education?  **Summative (Last session):** Educator project. |
|  | Describe the ideal self as an educator. | **Individual activity (15 minutes):** who am I? Do I want to impart my knowledge, skills, and attitudes? What knowledge, skills, attitudes do I want to impart? To whom? how?  How can I best be involved in dermatology education and broader medical education? | Instrument unique to activity.  Powerpoint presentation for prompts/Projector | **Formative:** Fill document, receive individual verbal feedback from facilitator.  **Summative (Last session):** Educator project. |
|  | Conclusions and action plan (5 minutes) | | | |

| **2.2 Module 2:**  Fulfilling the teacher role *(Kelcheterman’s model: Self-esteem and task perception)* | | | **130 minutes** | **December 2019** |
| --- | --- | --- | --- | --- |
|  | **Intended learning outcome (ILO)** | **Instructional design** | **Material** | **Assessment** |
| To prepare dermatology trainees for their teaching roles. | Introductions and signposting (10 minutes) | | | |
|  | Discuss four theories of learning that inform residency education: Behaviorism, Cognitive Load theory, Constructivism, and Complexity Theory. | **Brief didactic activity:** 10 minutes  **Small group activity (5 persons):** Each group brainstorms for 10 minutes then **large group discussion** 10 minutes. **Main point:** How are we taught in the dermatology residency program? ***Prompts***: What learning activities take place?(List 3) What beliefs inform such activities? | Pop-up board and markers for each group  Powerpoint presentation/Projector | **Formative:** Give small group verbal feedback during discussions; give verbal group feedback on performance after large discussion.  Topic: Where does knowledge in the field of dermatology come from?  **Summative (Last session):** Educator project. |
|  | Critique teaching strategies in residency education. | **Small group activity (5 persons):** Each group brainstorms for 10 minutes then **large group discussion** 10 minutes. **Main point:** Continue from previous activity: Are the teaching strategies currently used beneficial? ***Prompts***: How would we improve them? What would we do differently? |  |  |
|  | 10 minute break | | | |
|  | Describe the ideal self as a teacher. | **Brief didactic activity:** 10 minutes  **Individual activity (15 minutes):** Who am I as a teacher? How do I like to teach? What do I like to teach? What does the institute want me to teach? What strategies do I gravitate towards? What teaching strategies do I need to learn? | Notepad and pen  Powerpoint presentation/Projector | **Formative:** Fill document, receive individual verbal feedback from facilitator.  Topic: How do you teach to and with others?  **Summative (Last session):** Educator project. |
|  | Devise an action plan for personal growth as a teacher. | **Brief didactic activity:** 10 minutes  **Individual Activity (15 minutes):** Where am I as a teacher? Where do I need to be? Use of Gibb’s reflective cycle: Description, feeling, evaluation, conclusions, and actions.  **Work in pairs (10 minutes):** critique each other's work. | Instruments unique to activity.  Powerpoint presentation for prompts/Projector | **Formative:** Fill document, receive individual verbal feedback from facilitator.  **Summative (Last session):** Educator project. |
|  | Conclusions and action plan (5 minutes) | | | |

| **2.3 Module 3:**  How is knowledge made? *(Kelcheterman’s model: future prospects)* | | | **110 minutes** | **January 2019** |
| --- | --- | --- | --- | --- |
|  | **Intended learning outcome (ILO)** | **Instructional design** | **Material** | **Assessment** |
| To engage dermatology trainees in the knowledge production of the field. | Introductions and signposting (10 minutes) | | | |
|  | Discuss the concept of knowledge production in the field of dermatology. | **Brief didactic activity:** 10 minutes  **Small group activity (5 persons):** Each group brainstorms for 10 minutes then **large group discussion** 10 minutes. **Main point:** Where does knowledge in the field of dermatology come from? ***Prompts***: Books, journals, universities, research centers? Saudi versus non-Saudi? Scientists, practitioners, patients? English vs Arabic? | Pop-up board and markers for each group  Powerpoint presentation/Projector | **Formative:** Give small group verbal feedback during discussions; give verbal group feedback on performance after large discussion.  **Summative (Last session):** Educator project. |
|  | Discuss the concept of knowledge dissemination in the field of dermatology. | **Small group activity (5 persons):** Each group brainstorms for 10 minutes then **large group discussion** 10 minutes. **Main point:** Continue from previous activity: How is dermatological knowledge managed and distributed? ***Prompts***: Medical Colleges? Residency program, Saudi commission, saudi society for dermatology? The Saudi journal? |  |  |
|  | 10 minute break | | | |
|  | Explain the role of an educator in knowledge production and dissemination. | **Small group activity (5 persons):** Each group brainstorms for 10 minutes then **large group discussion** 10 minutes. **Main point:** As an educator, what is your role in knowledge production and dissemination? ***Prompts***: by being a teacher? Presentations skills, lectures, workshops? By conducting research? By cross-pollinating with other fields? By translating? | Notepad and pen  Powerpoint presentation/Projector | **Formative:** Fill document, receive individual verbal feedback from facilitator.  **Summative (Last session):** Educator project. |
|  | Describe the ideal self as knowledge producer and disseminator. | **Individual Activity (15 minutes):** Have I personally made knowledge? What is it? Am I a knowledge disseminator? Why? How?  **Work in pairs (10 minutes):** critique each other's work. | Instruments unique to activity.  Powerpoint presentation for prompts/Projector | **Formative:** Fill document, receive individual verbal feedback from facilitator.  **Summative (Last session):** Educator project. |
|  | Conclusions and action plan (5 minutes) | | | |

| **2.4 Module 4:** Education: a team sport (Kelchterman’s model: job motivation) | | | **130 minutes** | **February 2019** |
| --- | --- | --- | --- | --- |
|  | **Intended learning outcome (ILO)** | **Instructional design** | **Material** | **Assessment** |
| To increase collegiality in the field of dermatology. | Introductions and signposting (10 minutes) | | | |
|  | Differentiate personal, interpersonal, and cultural meaning of collegiality. | **Brief didactic activity:** 10 minutes  **Small group activity (5 persons):** Each group brainstorms for 10 minutes then **large group discussion** 10 minutes. **Main point:** What does collegiality mean? ***Prompts***: to me? What does it mean to us? In dermatology residency? In dermatology field? | Pop-up board and markers for each group  Powerpoint presentation/Projector | **Formative:** Give small group verbal feedback during discussions; give verbal group feedback on performance after large discussion.  **Summative (Last session):** Educator project. |
|  | Discuss collegiality in medical education and practice. | **Small group activity (5 persons):** Each group brainstorms for 10 minutes then **large group discussion** 10 minutes. **Main point:** collegiality in medical education and practice ***Prompts***: being an assessor? In giving feedback? Role-modelling? Mentoring? During medical school? Residency program? 10 years after postgraduate training? Who is at the other end of these activities? |  |  |
|  | 10 minute break | | | |
|  | Discuss manifestations of collegiality in dermatology field. | **Small group activity (5 persons):** Each group brainstorms for 10 minutes then **large group discussion** 10 minutes. **Main point:** when is collegiality necessary? ***Prompts***: How to give feedback? How to conduct a lecture? How to be more interactive? Why? | Notepad and pen  Powerpoint presentation/Projector | **Formative:** Fill document, receive individual verbal feedback from facilitator.  **Summative (Last session):** Educator project. |
|  | Explain the role of an educator in establishing and maintaining collegiality. | **Brief didactic activity:** 10 minutes  **Individual Activity (15 minutes):** AS an educator, what is my role in collegiality? How do I maintain it?  **Work in pairs (10 minutes):** critique each other's work. | Instruments unique to activity.  Powerpoint presentation for prompts/Projector | **Formative:** Fill document, receive individual verbal feedback from facilitator.  **Summative (Last session):** Educator project. |
|  | Conclusions and action plan (5 minutes) | | | |

**2.5 Summative assessment**

Oriented by Kirkpatrick’s Evaluation Model, we summatively assessed the learning of residents. We addressed the first two levels: *reaction* and *learning*. Because levels 3 and 4, *behavior change and impact on patients*, require time and resources, we were not able to address them. We mention them here to signal the importance of addressing all 4 levels, as levels 3 and 4 are important for sustainability of the suggested program and residents identity development (See table below). We hope to embark on addressing these levels in future iterations of this program.

| **Kirkpatrick’s evaluation model level** | **Description of level** | **Assessment method** |
| --- | --- | --- |
| Reaction | How learners felt about teaching. | Oral feedback after each session. |
| Learning | How learners' knowledge has changed after a program. | -Written reflections after each session.  -Final oral presentation. |
| Behavior | The degree to which learners applied the knowledge | Not applied |
| Results | The impact of the enhanced learner skill on their environment. | Not applied |

Through oral presentations in the last session, prompts from the written reflections were revisited and residents were asked to address them along with the large group. Residents were evaluated by the program directors and instructor of the workshop.
