# Supplementary material for: Dermatology residents as educators: a qualitative study of identity formation
Source: BMC Med Educ. 2023 Mar 30;23:199. doi: 10.1186/s12909-023-04186-4 (PMC10061385; doi:10.1186/s12909-023-04186-4)
Supplement: Supplementary file 2 — Supplementary Material 2 [file 12909_2023_4186_MOESM2_ESM.docx]

**Appendix B: Interview guide**

**Title of the study**: Dermatology residents as educators: a qualitative study on identity construction

Version 3

**Welcome**

• Welcome the participant

*Welcome and thank you for agreeing to participate in this project. We recognize your busy schedule and appreciate your cooperation.*

**Introduction**

• Name and background

*My name is (State name) and I'm an (State position and employer).*

• State the purpose of the interview

*The interview will revolve around your views and experience as an educator as well as your experiences or experiences of others you may have witnessed.*

• Language

*The interview will be conducted in English. However, feel free to express yourself in Arabic if you need to do so.*

• Queries

*Do you have any questions you would like to ask before we start?*

**Anonymity and right to withdraw**

• The interview will be audio-recorded but your identity will remain anonymous.

• The audio files will be transcribed after the interview.

• You will be assigned a participant number to protect your identity.

• The audio files will be kept in a secure server abiding by research governance policies.

• You have the right to withdraw from the interview at any time with no penalty to yourself.

• You do not have to answer questions that make you feel uncomfortable. You may find some

of these issues personal. If this is the case, feel free to inform the researcher.

• Please refrain from mentioning specific dates, names of places or individual

| **Research question** | **Study objective** | **Kelchterman’s conceptual model related dimensions** | **Question** | **Prompts** |
| --- | --- | --- | --- | --- |
| How do dermatology residents form identities as educators? | Explore educator identity construction amongst dermatology residents. | Self-image and task perception | Please describe your experiences as an educator during your career? | During your undergraduate experience?  During your residency? |
|  |  | Self-esteem | Could you share a memorable  experience you have had as an educator? | Who was there?  Where was it?  How did it impact you?  How did that change you? |
|  | Examine how dermatology residents define education. | Self-image and task perception | What does the term education mean to you? | How do you see yourself as an educator?  How is education defined in your context? |
|  | Identify factors that support educator identity construction in dermatology residency programs. | Job motivation and self esteem | What factors supported your growth as an educator? | self -motivated?  Role-models?  Formal education on this role during residency education?  How did they influence your growth?  What values inform your educator role?  Does the work/learning influence it? |
|  | Identify factors that discourage educator identity construction. | Job motivation and self-esteem | What factors discourage your growth as an educator? | self -motivated?  Role-models?  Formal education on this role during residency education?  How did they influence your growth?  What changed? |
| What is the role of professional development programs in dermatology residents’ growth as educators? | Explore the role of professional development programs in dermatology residents’ growth as educator. | Future prospective | How do you view your future role as an educator? | Is it self-directed individual effort?  Do others influence your future participation as an educator?  What future role would you take as an educator in dermatology? |

**Conclusion**

• Acknowledgment

*Thank you for taking part in this interview. Your participation greatly enriched the study and we appreciate your contribution.*

• Reiterate important information

*After analysis of the data, we will send a copy of the report to everyone who requests to receive one on the consent form. Those will be given the opportunity to comment on the result before they are submitted for publication.*

*We assure your anonymity and that of your workplace is of utmost importance throughout all stages of this study.*

This project has been reviewed and approved by the Security Forces Hospital Institutional Review Board. IRB registration number **H-01-R-069**.
