# Supplementary material for: Dermatology residents as educators: a qualitative study of identity formation
Source: BMC Med Educ. 2023 Mar 30;23:199. doi: 10.1186/s12909-023-04186-4 (PMC10061385; doi:10.1186/s12909-023-04186-4)
Supplement: Supplementary file 3 — Supplementary Material 3 [file 12909_2023_4186_MOESM3_ESM.docx]

| **How do dermatology residents form identities as educators?**  (n=179) | | |
| --- | --- | --- |
| **Theme:** Defining education (n=50) | | |
| **Sub-theme** | **Definition** | **Keltcherman’s corresponding domain** |
| Definitions of education (n=14) | This theme encompassed any definition offered by participant for the construct of education. | Task perception |
| Attributes of a good educator (n=23) | This sub-theme encompassed any description or explanation of personal characteristics related to education. | Self-image |
| Aspirations of being a better educator (n=13) | This sub-theme included residents’ aspirations of being a better educator and ways to enhance educational practice. | Self-esteem |
| **Theme:** Process of education (n=10) | |  |
| Learning resources (n=2) | In this sub-theme, residents described learning resources used and recommended in dermatology. | Task perception |
| Paying it forward (n=4) | In this sub-theme, residents described the process of education as a voluntary act and instead of expecting receiving something in return they expect the same person to educate others. | Job motivation |
| Context-based (n=4) | In this sub-theme, residents viewed the process of education as context based, whether in residency education or outside of it, in the clinic or in the lecture hall. | Task perception |
| **Theme:** Educator identity formation (n=119) | |  |
| Weak identity (n=12) | In this sub-theme, resident conceptualized shied away from their educator identities, expressing lack of educator role. | Self-image |
| Educator as leader (n=7) | In this sub-theme, residents described their educator identity as one of leadership or one of its aspects e.g. influence, power. | Self-image |
| Educator as simplifier (n=17) | In this sub-theme, residents described their educator identity as one of simplifying information for others to understand. | Task perception |
| Educator as knowledge curator (n=5) | In this sub-theme, residents described their educator identity as custodians of knowledge and in charge of selecting what to present to learners. | Task perception |
| Educator as team member (n=25) | In this sub-theme, residents described their educator identity as being part of a team, rather than an individual act. | Job motivation |
| Educator as community advocate (n=12) | In this sub-theme, residents described their educator identity as someone who lobbies for the development of the larger community and takes responsibility for the field of dermatology. | Task perception |
| Educator as self-motivated (n=6) | In this sub-theme, residents described their educator identity as someone who is internally motivated to educate others. | Job-motivation |
| Educator as learner (n=20) | In this sub-theme, residents described their educator identity as someone who is a learner prior to being an educator, engaging continuously in self-directed learning. | Self-image |
| Educator as collaborator (n=15) | In this sub-theme, residents described their educator identity as someone who works with others to achieve positive outcomes. | Job motivation |

| **What is the role of professional development programs in residents’ development as educators?** *(n=20)* | | |
| --- | --- | --- |
| **Theme:** Resident as educator program (n=46) | | |
| **Sub-theme** | **Definition** | **Keltcherman’s corresponding domain** |
| Individual lifelong effort (n=12) | Here residents viewed preparation for their role as educators to be a personal responsibility that should be addressed though self-directed learning. | Future perspectives |
| Collegiality (n=15) | In this sub-theme encompasses any description provided by residents regarding interpersonal efforts to provide education as well as opportunities for peer learning. | Future perspectives |
| Organizational role (n= 19) | This sub-theme encompassed residents’ opinions regarding the role of the residency program and the field of dermatology in developing residents in their roles as educators. | Future perspectives |
